# Associations of the BRAF^V600E^ Mutation with Sonographic Features and Clinicopathologic Characteristics in a Large Population with Conventional Papillary Thyroid Carcinoma

**DOI:** 10.1371/journal.pone.0110868

**Published:** 2014-10-22

**Authors:** Ah Young Park, Eun Ju Son, Jeong-Ah Kim, Ji Hyun Youk, Yun Joo Park, Cheong Soo Park, Hang Seok Chang

**Affiliations:** 1 Department of Radiology and Research Institute of Radiological Science, Yonsei University College of Medicine, Gangnam Severance Hospital, Seoul, Republic of Korea; 2 Department of Radiology, Soonchunghyang University Hospital, Soonchunghyang University College of Medicine, Seoul, Republic of Korea; 3 Department of Surgery, Thyroid Cancer Center, Yonsei University College of Medicine, Gangnam Severance Hospital, Seoul, Republic of Korea; Universite Libre de Bruxelles (ULB), Belgium

## Abstract

**Objective:**

To evaluate the association of the BRAF^V600E^ mutation with sonographic features and clinicopathologic characteristics in a large population with conventional papillary thyroid carcinoma (PTC).

**Methods:**

We retrospectively reviewed the sonographic features, clinicopathologic characteristics, and presence of the BRAF^V600E^ mutation in 688 patients who underwent thyroidectomy for conventional PTC between January and July 2010 at a single institution. The incidence of the BRAF^V600E^ mutation was calculated. The sonographic features and clinicopathologic characteristics were compared between BRAF-positive and BRAF-negative patients. BRAF-positive patients were subdivided into those with papillary thyroid microcarcinoma (the PTMC group) and those with PTC larger than 10 mm (the PTC>10 mm group), and their sonographic features were compared.

**Results:**

The BRAF^V600E^ mutation was detected in 69.2% of patients (476 of 688). Sonographic features were not significantly different between BRAF-positive and BRAF-negative PTC, nor between PTMC and PTC>10 mm groups. The BRAF^V600E^ mutation was associated with male sex (*P* = 0.028), large tumor size, extrathyroidal extension, central and lateral lymph node metastasis, and advanced tumor stage (*P*<0.0001).

**Conclusion:**

The BRAF^V600E^ mutation was significantly associated with several poor clinicopathologic characteristics, but was not associated with sonographic features, regardless of tumor size. We recommend that patients with a thyroid nodule with any suspicious sonographic feature undergo preoperative BRAF^V600E^ testing for risk stratification and to guide the initial surgical approach in PTC.

## Introduction

Papillary thyroid carcinoma (PTC) is the most common thyroid cancer, accounting for 85-90% of cases, with an increasing incidence globally [1], [2]. The development of high-resolution ultrasonography (US) has contributed to the detection and diagnosis of PTC with high specificity, but relatively low sensitivity [3], [4]. US-guided fine-needle aspiration (US-FNA) biopsy is a standard tool for diagnosing thyroid malignancies preoperatively with high specificity. Its major limitation, however is the 15–25% rate of indeterminate cytology (Bethesda category I-nondiagnostic or category III- atypia of undetermined significance or follicular lesion of undetermined significance) [5].

Various genetic analyses have improved the diagnostic performance of US-FNA. Above all, the B-type Raf kinase (BRAF) mutation has received the most attention in recent years because of its high prevalence and high specificity for PTC. The BRAF mutation induces aberrant activation of the mitogen-activated protein kinase pathway, which plays a fundamental role in cell proliferation, differentiation, and apoptosis, finally resulting in tumorigenesis [1], [6], [7]. The T1799A point BRAF mutation is the most common mutation found in the BRAF gene, accounting for more than 90% of mutations. It causes a V600E amino acid change in the BRAF protein, resulting in a BRAF^V600E^ mutation that occurs exclusively in PTC with a prevalence ranging from 29 to 83% [1], [7]. Several previous reports have demonstrated that BRAF^V600E^ mutation testing may enhance the diagnostic accuracy of US-FNA for PTC [8]–[13].

The roles of the BRAF^V600E^ mutation have been found to include the down-regulation of tumor suppressor genes, up-regulation of tumor-promoting molecules, and resulting promotion of tumor growth, angiogenesis, tissue invasion and metastasis [6]. In addition, a number of reports have demonstrated a direct association of the BRAF^V600E^ mutation with poor prognostic factors such as extrathyroidal extension, lymph node and distant metastases, advanced tumor stage, and tumor recurrence, although it remains controversial [14]–[28]. Several studies have also found a positive correlation between the BRAF^V600E^ mutation and suspicious sonographic features of thyroid nodules [11]–[29]. However, only a few reports have been published on the association between the BRAF^V600E^ mutation and sonographic features in PTC; findings have varied according to tumor size [2], [13], [30], [31].

Therefore, we evaluated the association of the BRAF^V600E^ mutation with sonographic features and clinicopathologic characteristics in a large-scale study population with conventional PTC.

## Materials and Methods

The institutional review board of Gangnam Severance hospital approved of this retrospective observational study and required neither patient approval nor informed consent for our review of patients’ images and records. However, written informed consent was obtained from all patients for US-FNA and BRAF^V600E^ mutation analysis prior to each procedure as a daily practice.

### Patients

Our hospital’s institutional review board approved this retrospective observational study and waived the requirement for informed consent. Between January and July 2010, 939 consecutive patients underwent thyroidectomy and were diagnosed with conventional PTC at our institution. Of these, 251 patients were excluded for the following reasons: patient refusal of BRAF^V600E^ mutation analysis of the surgical specimen (n = 87), lack of preoperative US at our institution (n = 161) and inability to identify the lesion on US (n = 3). A total of 688 patients were included in this study (553 women and 135 men; mean age, 45 years; range, 17–83).

All patients were diagnosed preoperatively with malignancy by US-FNA at our institution (n = 222) or outside clinics (n = 466). US-FNA was performed on all suspicious thyroid nodules larger than 5 mm, and on nodules smaller than 5 mm at the patient’s or clinician’s request. Cytologic results were as follows: 531 papillary carcinomas, 147 cases suspicious for papillary carcinoma, 8 cases with atypia of undetermined significance, 1 benign follicular nodule, and 1 lymphocytic thyroiditis. Eight atypical lesions were surgically removed due to sonographic features compatible with papillary carcinoma (n = 7) or histologic confirmation of malignancy through core biopsy (n = 1). Two benign cases were surgically removed due to sonographic feature compatible with papillary carcinoma and lymph node metastasis on US-FNA.

All patients received curative surgery with either total thyroidectomy (n = 517) or near-total thyroidectomy (n = 171). Prophylactic or therapeutic central-compartment neck dissection was performed for all patients. Lateral compartmental lymph node dissection was performed for patients with US-FNA-proven or clinically suspicious lateral cervical lymphadenopathy (n = 61).

### Ultrasound examination

US images were obtained using either HDI5000 or IU22 ultrasound scanners (Philips Healthcare, Bothell, WA) equipped with a 7.5–12 MHz linear array transducer. Four radiologists who specialize in thyroid US with between 1 and 12 years of experience (E.J.S., J.A.K., J.H.Y., and A.Y.P.) performed all US examinations before BRAF^V600E^ mutation analysis was conducted. Two radiologists (A.Y.P and E.J.S, with 1 and 12 years of experience, respectively) blinded to BRAF status retrospectively analyzed the following sonographic features in consensus: tumor size, composition (solid or cystic), echogenicity with respect to the thyroid parenchyma and strap muscle (hyperechoic, isoechoic, hypoechoic or markedly hypoechoic), margin (circumscribed, microlobulated or irregular), calcifications (microcalcification, macrocalcification, or negative), and shape (parallel or nonparallel). US findings of microcalcification, irregular or microlobulated margin, marked hypoechogenicity, and nonparallel shape are considered indicative of malignancy. Final assessment category was classified according to the number of suspicious features, which was based on the modified thyroid imaging reporting and data system (TIRADS) suggested by Kwak et al. (4): probably benign (no suspicious US feature), low suspicion for malignancy (1 suspicious feature), intermediate suspicion for malignancy (2 suspicious feature), moderate suspicion for malignancy (3 suspicious features) or highly suggestive of malignancy (4 suspicious features).

### Clinicopathologic data analysis

Medical records were reviewed to determine sex and age at diagnosis. Pathology reports were reviewed for the following data: tumor size, presence of BRAF^V600E^ mutation, multifocality, extrathyroidal extension, and central or lateral lymph node metastasis. TNM stage was assigned based on the *AJCC Cancer Staging Manual* 7^th^ edition [32]. Patients were divided into two groups according to pathologic tumor size less than 10 mm (papillary thyroid microcarcinoma, PTMC group) or greater than 10 mm (PTC>10 mm group).

### BRAF^V600E^ mutation analysis

DNA was extracted from 10-mm-thick sections of paraffin blocks using the QIAamp DNA FFPE Tissue Kit (Qiagen, Hilden, Germany). DNA was extracted only from the marked tumor tissue. The presence of a BRAF mutation was evaluated by polymerase chain reaction-restriction fragment length polymorphism (PCR-RFLP) or direct sequencing. For PCR-RFLP, a 50-μL PCR mixture was composed of extracted DNA at 100 ng/mL, 5 U Taq polymerase, 0.25 mM dNTP, 20 pmol of primers, and 10× Ex Taq buffer. To examine the BRAF exon 15, a primer that created a restriction site for the BspE1 enzyme was designed. The PCR conditions were as follows: denaturation at 95°C for 10 minutes, followed by 40 cycles at 94°C for 30 seconds, 45°C for 30 seconds, 72°C for 30 seconds, and a final extension step at 72°C for 10 minutes. The PCR product was purified with the MinElute PCR purification kit (Qiagen), digested with 10 units of BspE1 (Takara, Tokyo, Japan) and electrophoresed in a 4% agarose gel containing ethidium bromide. The stained gel was photographed using an ultraviolet light transilluminator. For direct sequencing, BRAF exon 15, which contains the codon encoding the V600E mutation, was amplified. The PCR conditions were as follows: denaturation at 94°C for 5 minutes, followed by 40 cycles at 94°C for 20 seconds, 56°C for 30 seconds, 72°C for 30 seconds, and a final extension step at 72°C for 5 minutes. PCR products were purified using the Exo I/SAP Clean-Up protocol (Hilden, Germany), and direct DNA sequencing was performed using the 3730 Big Dye Terminator v 3.1 Sequencing Standard (Applied Biosystems, Foster City, CA).

### Statistical analysis

The incidence of the BRAF^V600E^ mutation was calculated. Sonographic features and clinicopathologic characteristics were compared between the patients with and without the BRAF^V600E^ mutation. The correlation between sonographic features and the BRAF^V600E^ mutation was also evaluated in the PTMC and PTC>10 mm groups. The chi-squared or Fisher’s exact test was used for categorical variables and the Student’s t-test was used for continuous variables. Multivariate logistic regression analysis was performed for the association of the BRAF^V600E^ mutation with sonographic features in patients with PTC. Statistical significance was accepted for *P*-values less than 0.05. All statistical analysis was performed using SPSS version 19.0 (SPSS, Inc., Chicago, IL).

## Results

Of the 688 patients with PTC, 514 (71.4%) had PTMC, 174 (28.6%) had PTC>10 mm, and 476 (69.2%) had the BRAF^V600E^ mutation. The incidence of the BRAF^V600E^ mutation was significantly higher in the PTC>10 mm group than the PTMC group (81.0% *vs*. 65.2%, *P*<0.0001)

### Sonographic features

BRAF-positive PTC were significantly larger than BRAF-negative PTC on US (10.8±6.8 mm *vs*. 8.4±4.9 mm, *P*<0.0001). There were no significant differences in sonographic features between BRAF-positive and BRAF-negative PTC, including composition, echogenicity, margin, calcification, shape or final assessment (Table 1). Multivariate logistic regression analysis also showed no association between the BRAF^V600E^ mutation with suspicious sonographic features (Table 2).

**Table 1:** Association of the BRAF^V600E^ mutation with sonographic features in patients with PTC.

		PTMC (n = 514)			PTC>10 mm (n = 174)			All PTC (n = 688)		
		BRAF-positive (n = 335)	BRAF-negative (n = 179)	*P-*value	BRAF-positive (n = 141)	BRAF-negative (n = 33)	*P-*value	BRAF-positive (n = 476)	BRAF-negative (n = 212)	*P-*value
Sonographic size (mm)*		8.0±3.8	7.1±2.7	.004	17.7±7.4	15.6±7.5	.164	10.8±6.8	8.4±4.9	<.0001
Composition	Solid	331 (65.2)	177 (34.8)	1.000	133 (81.6)	30 (18.4)	.439	464 (69.2)	207 (30.8)	.899
	Cystic	4 (66.7)	2 (33.3)		8 (72.7)	3 (27.3)		12 (70.6)	5 (29.4)	
Echogenicity	Hyper-/Isoechoic	7 (63.6)	4 (36.4)	.716	10 (66.7)	5 (33.3)	.321	18 (65.4)	8 (34.6)	.673
	Hypoechoic	111 (67.7)	53 (32.3)		48 (81.4)	11 (18.6)		159 (71.3)	64 (28.7)	
	Markedly hypoechoic	217 (64.0)	122 (36.0)		83 (83.0)	17 (17.0)		300 (68.3)	139 (31.5)	
Margin	Circumscribed	7 (63.6)	4 (36.4)	.807	5 (71.4)	2 (28.6)	.770	12 (66.7)	6 (33.3)	.524
	Microlobulated	34 (69.4)	15 (30.6)		25 (83.3)	5 (16.7)		59 (74.7)	20 (25.3)	
	Irregular	294 (64.8)	160 (35.2)		111 (81.0)	26 (19.0)		405 (68.5)	186 (31.5)	
Calcifications	Microcalcifications	223 (66.2)	114 (33.8)	.624	114 (81.4)	26 (18.6)	.717	337 (70.6)	140 (29.4)	.351
	Macrocalcifications	22 (68.8)	10 (31.2)		11 (73.3)	4 (26.7)		33 (70.2)	14 (29.8)	
	Negative	90 (62.1)	55 (37.9)		16 (84.2)	3 (15.8)		106 (64.6)	58 (35.4)	
Shape	Parallel	238 (67.6)	114 (32.4)	.087	84 (84.8)	15 (15.2)	.140	322 (71.4)	129 (28.6)	.083
	Nonparallel	97 (59.9)	65 (40.1)		57 (76.0)	18 (24.0)		154 (65.0)	83 (35.0)	
Final assessment†	Probably benign	1 (50.0)	1 (50.0)	.500	1 (33.3)	2 (66.7)	.065	2 (40.0)	3 (60.0)	.114
	Low suspicion for malignancy	26 (70.3)	11 (29.7)		9 (100)	0 (0)		35 (76.1)	11 (23.9)	
	Intermediate suspicion for malignancy	66 (58.4)	47 (41.6)		29 (72.5)	11 (27.5)		95 (62.1)	58 (37.9)	
	Moderate concern for malignancy	120 (66.7)	60 (33.3)		58 (81.7)	13 (18.3)		178 (70.9)	73 (29.1)	
	Highly suggestive of malignancy	122 (67.0)	60 (33.3)		44 (86.3)	7 (13.7)		166 (71.2)	67 (28.8)	

Data are raw numbers, percentages are in parentheses.

Abbreviations: PTMC, papillary thyroid microcarcinoma; PTC, papillary thyroid carcinoma.

^*^ Numbers present the mean±standard deviation.

† Final assessment was classified according to the number of suspicious US features, which was based on the modified thyroid imaging reporting and data system (TIRADS) suggested by Kwak et al. (4).

**Table 2:** Multivariate analysis of the association of the BRAF^V600E^ mutation with sonographic features in patients with PTC.

	Odds ratio (95% CI)	*P*-value
Sonographic ize (mm)	1.102 (1.058–1.148)	<.0001
Markedly hypoechoic vs Hyper-/Iso-/Hypoechoic	1.159 (0.815–1.649)	.412
Irregular/microlobulated vs Circumscribed	0.974 (0.341–2.781)	.961
Microcalcifications vs Negative/Macrocalcification	1.057 (0.726–1.539)	.771
Non-parallel vs Parallel	0.603 (0.419–0.867)	.006

95% CI = 95% confidence interval.

BRAF-positive PTMC were significantly larger than BRAF-negative PTMC (8.0±3.8 mm *vs*. 7.1±2.7 mm, *P* = 0.004). However, there was no significant difference in size according to the BRAF^V600E^ mutation in the PTC<10 mm group (17.7±7.4 mm *vs*. 15.6±7.5 mm, *P* = 0.164). In addition, there were no significant differences in other sonographic features according to the BRAF^V600E^ mutation in either the PTMC or PTC>10 mm group (Table 1). Large lesion size was an independent predictive factor for BRAF positivity on multivariate analysis (Odds ratio [95% confidence interval]; 1.102 [1.058–1.148], *P*<.0001) (Table 2).

### Clinicopathologic characteristics

Compared to BRAF-negative patients, BRAF-positive patients were more likely to be male (21.8% *vs*. 14.6%, *P* = 0.028) and to have a larger pathologic tumor size (9.1±5.9 mm *vs*. 6.6±4.2 mm, *P*<0.0001).

The associations between the BRAF^V600E^ mutation and clinicopathologic characteristics are summarized in Table 3. BRAF-positive PTC presented more frequently with extrathyroidal extension compared to BRAF-negative PTC (64.1% *vs*. 43.9%, *P*<0.0001). Lymph node metastases (central/lateral) were significantly more frequent in BRAF-positive than BRAF-negative PTC (31.1/10.9% *vs*. 17.9/5.2%, *P*<0.0001). Also, BRAF-positive PTC were more likely to present at a higher (III/IV) TNM stage compared to BRAF-negative PTC (31.1/42.2% *vs*. 29.2/23.1%, *P*<0.0001). There were no significant differences in age or lesion multiplicity according to BRAF status.

**Table 3:** Association of the BRAF^V600E^ mutation with clinicopathologic characteristics of patients with PTC.

		BRAF-positive PTC (n = 476)	BRAF-negative PTC (n = 212)	*P-*value
Age*		45.2±11.2	45.4±11.2	.784
Sex	Female	372 (78.2)	181 (85.4)	.028
	Male	104 (21.8)	31 (14.6)	
Pathologic size (mm)*		9.1±5.9	6.6±4.2	<.0001
	PTMC	335 (70.4)	179 (84.4)	<.0001
	PTC>10 mm	141 (29.6)	33 (15.6)	
Multiplicity	Negative	321 (67.4)	151 (71.2)	.323
	Positive	155 (32.6)	61 (28.8)	
Extrathyroidal extension	Negative	171 (35.9)	119 (56.1)	<.0001
	Positive	305 (64.1)	93 (43.9)	
Lymph node metastasis	Negative	276 (58.0)	163 (76.9)	<.0001
	Central	148 (31.1)	38 (17.9)	
	Lateral	52 (10.9)	11 (5.2)	
Tumor stage	I	125 (26.3)	101 (47.6)	<.0001
	II	2 (0.4)	0 (0)	
	III	148 (31.1)	62 (29.2)	
	IV	201 (42.2)	49 (23.1)	
	III/IV	349 (73.3)	111 (52.3)	<.0001

Data are raw numbers, percentages are in parentheses.

Abbreviations: PTMC, papillary thyroid microcarcinoma; PTC, papillary thyroid carcinoma.

* Numbers present the mean±standard deviation.

## Discussion

Previous meta-analyses have published values for the overall prevalence of the BRAF^V600E^ mutation ranging from 29 to 83% [7], [14]–[16]. This wide range may be due to variations in PTC subtype, subjects’ geographical backgrounds, and research methodology. Korea appears to have a relatively high frequency of the BRAF^V600E^ mutation, ranging from 52 to 83% [14], [15], [23], [25], [33]. Our large-scale study of conventional PTC in Korea also found a relatively high prevalence of 69.2%.

In regards to the association between the BRAF mutation and sonographic features of PTC, two recent studies reported no significant difference in sonographic features between BRAF-positive and BRAF-negative PTC [2], [13]. A Korean study on PTMC alone also reported no significant difference [31]. In contrast, a recent study on 115 patients with PTC larger than 10 mm found that BRAF-positivity was associated with suspicious sonographic findings and the number of suspicious features has positive correlation with the risk of BRAF positivity [30]. The authors suggested that difficulties in accurate sonographic characterization of small PTC confounded studies including small PTC. However, our study found no significant difference in any sonographic feature between BRAF-positive and BRAF-negative PTC in the PTC>10 mm group as well as PTMC group. Final assessment category classified according to the number of suspicious features was also not significantly different between BRAF-positive and BRAF-negative PTC, although the PTC>10 mm group had more tendency in correlation between BRAF-positivity and final assessment category than PTMC group did (*P*-value, 0.065 *vs*. 0.500). We assume that such discrepancy might result from different study population between ours and the above-mentioned study. Most suspicious sonographic features used in both studies are oriented to diagnosis of conventional PTC, therefore only inclusion of conventional PTC in our study may predispose little distinction of sonogrpahic features between BRAF-positive and BRAF-negative PTC. The result of our study suggests that there is no specific sonographic feature to be an indication for performing additional BRAF^V600E^ mutation analysis to FNA for the thyroid nodules suspicious of PTC.

The tumor size of BRAF-positive PTC, both sonographic and pathologic, were larger than that of BRAF-negative PTC. Previous studies have also reported an association between the BRAF mutation and large tumor size, which suggests the possibility that the mutation induces tumor progression and aggressiveness [23], [26], [34], [35]. However, other studies have reported conflicting results [2], [14], [18], [22], [33], indicating that the relationship between the BRAF mutation and tumor size remains controversial. Our results also showed that the BRAF mutation was more frequent in men, consistent with previous studies [26], [36].

In respect to the relationship between the BRAF mutation and clinicopathologic PTC characteristics, many studies have reported that one or more high-risk clinicopathologic parameters were associated with the BRAF mutation [14]–[28]. Two recent meta-analyses that included 5655 and 2470 PTC patients, respectively, found a significant association between the BRAF mutation and lymph node metastasis, extrathyroidal extension, advanced tumor stage and recurrence [15], [16]. In addition, a recent study with a median 15 years of follow-up demonstrated that the BRAF mutation was related to advanced tumor stage, vascular invasion, and mortality [19]. Our results also revealed a relationship between BRAF-positive PTC and high-risk clinicopathologic characteristics, including extrathyroidal extension, lymph node metastasis and advanced tumor stage. On the contrary, several studies employing multivariate analysis with adjustment of confounders found no such relationship with these characteristics [14], [33], [37]–[39]. Possible reasons for this discrepancy in results include variations in PTC subtypes, geographic or ethnic factors, scale of enrolled data, disease extent at the time of diagnosis, methodology of BRAF analysis, and the use of prophylactic central neck compartment dissection.

Our study has several unique strengths. First, this is the largest single-center study on conventional PTC, which limits participant heterogeneity and variations in tumor subtypes. Second, our finding that the BRAF mutation was associated with poor clinicopathologic parameters suggests the utility of preoperative BRAF analysis in risk stratification and surgical management, especially in cases of equivocal extrathyroidal extension or cervical node metastasis on preoperative US, or nondiagnostic result of cervical lymph node metastasis on US-FNA. Lastly, we analyzed the relationship between BRAF mutation and sonographic features in both PTMC and PTC>10 mm groups to establish the previous controversial results according to the tumor size and observed the result that there was no difference in sonographic features between the BRAF-positive PTC and BRAF-negative PTC in both PTMC and PTC>10 mm.

Our study has several limitations. First, this was a retrospective observational study, a design that prevents long-term follow-up or analysis of actual clinical outcomes. Second, we did not evaluate interobserver variability of PTMC sonographic features, despite known difficulty in accurate characterization of small tumors. Finally, we did not evaluate the Doppler or elastographic tumor findings, which can provide additional diagnostic information. Future areas of research include prospective long-term follow-up and Doppler or elastographic evaluation of PTC.

## Conclusion

The BRAF mutation was not associated with particular sonographic features in conventional PTC, regardless of tumor size. However, the mutation was significantly associated with poor clinicopathologic parameters including male gender, large tumor size, extrathyroidal extension, lymph node metastasis and advanced tumor stage. Our results suggest the utility of preoperative BRAF^V600E^ mutation analysis of thyroid nodules with any suspicious sonographic feature for risk stratification and determination of the initial surgical approach in PTC.
